# Life-Threatening Malnutrition in Very Severe ME/CFS

**DOI:** 10.3390/healthcare9040459

**Published:** 2021-04-14

**Authors:** Helen Baxter, Nigel Speight, William Weir

**Affiliations:** 1 25% ME Group, Troon KA10 6HT, UK; 2 Medical Advisor to 25% ME Group, Durham DH1 1QN, UK; speight@doctors.org.uk; 3 NHS Consultant Physician, London W1G 9PF, UK; wrcweir@hotmail.com

**Keywords:** Myalgic Encephalomyelitis (ME), Chronic Fatigue Syndrome (CFS), enteral feeding, Nasogastric Tube (NGT), Nasojejunal Tube (NJT), Percutaneous Endoscopic Gastrostomy (PEG), Total Parenteral Nutrition (TPN), Home Enteral Nutrition Service (HENS), Mast Cell Activation Disorder (MCAD)

## Abstract

Very severe Myalgic Encephalomyelitis (ME), (also known as Chronic Fatigue Syndrome) can lead to problems with nutrition and hydration. The reasons can be an inability to swallow, severe gastrointestinal problems tolerating food or the patient being too debilitated to eat and drink. Some patients with very severe ME will require tube feeding, either enterally or parenterally. There can often be a significant delay in implementing this, due to professional opinion, allowing the patient to become severely malnourished. Healthcare professionals may fail to recognize that the problems are a direct consequence of very severe ME, preferring to postulate psychological theories rather than addressing the primary clinical need. We present five case reports in which delay in instigating tube feeding led to severe malnutrition of a life-threatening degree. This case study aims to alert healthcare professionals to these realities.

## 1. Introduction

Some of the most severely affected ME patients experience serious difficulties in maintaining adequate nutrition and hydration and will require feeding enterally. There are a variety of mechanisms whereby nutritional difficulties arise and they are not uncommon in the most severe cases of ME/CFS. Perhaps the commonest is that the patient is just so debilitated that the sheer effort of eating and drinking is too much for them [1]. Another potential mechanism is that there are genuine difficulties with swallowing [2]; the causes for this are currently unclear. Finally, there may be problems lower down the alimentary tract such as gastroparesis, or features of malabsorption, with the additional possibility of Mast Cell Activation Disorder (MCAD). In these latter instances, enteral tube feeding may fail and recourse to Total Parenteral Nutrition (TPN) may be necessary. A lack of awareness by healthcare professionals of this problem may constitute a barrier to the patient receiving timely artificial nutrition (AN).

There is limited literature on this subject. The 2007 NICE Guidelines [3] make passing reference to the issue, as do the Paediatric Guidelines from The Royal College of Paediatricians and Child Health (RCPCH) in 2004 [4]. It is also mentioned in a recent paper on severe ME in young people [1]. Both ME/CFS [5] and nutrition [6] are poorly covered in the curriculum at medical schools.

The potential adverse consequences for the patient of their problem not being promptly recognized and responded to are considerable. There is often a significant delay in implementing tube feeding in patients experiencing difficulties obtaining nutrition and hydration. Tube feeding is often not instigated until the malnutrition becomes life threatening. Healthcare professionals seem to fail to recognize that the inability to eat and drink is a direct consequence of the severity of the ME, instead preferring to postulate psychological theories. Apart from the medical issues, it can be emotionally very upsetting for the patients to have their problems not recognized, or misdiagnosed.

As staff and volunteers with the UK’s 25% ME Group—the charity supporting people living with severe and very severe ME—we have become aware of these factors and have become very concerned by the clinical response both in the community and in acute settings.

This case report documents current NHS responses to patients in this situation, with the aim of raising awareness amongst healthcare professionals.

## 2. Methods

With the awareness of the difficulties people with very severe ME can have obtaining nutrition and hydration and the clinical response, the 25% ME Group devised a questionnaire for members who had experience of being enterally or parenterally fed. An invitation to participate was placed in the 25% ME Group charity’s newsletter, ‘The Quarterly’, in summer 2019, This was available either on paper, by post or via email. The questionnaire contained a range of questions such as age, reason for AN, type of AN, duration and an open-ended section for noting ‘any other relevant information’ (see Supplementary Materials).

Reasonable adjustments were put in place to maximize participation whilst attempting to minimize the likelihood of post exertional malaise. These included flexibility in the method of communication with patients and family/paid home worker staff and no fixed end date to return the questionnaire. Two of the patients’ ME was so severe that they were unable to read, write or type and in one case speak. A member of staff from 25% ME Group made direct contact with them and communicated via telephone, text or with their representative to gather information. As the member of staff has experience of talking to people with severe ME, she listened for signs of tiredness in the patient’s voice and terminated telephone calls at the first sign of fatigue.

Questionnaire responses provided a foundation; additional information was obtained using the patients’ preferred method of communication. From the information given, a series of anonymized case reports were developed. The case reports represent an opportunity sample.

## 3. Results

### 3.1. Case 1

This patient was diagnosed with severe ME as a child and has never fully recovered. She has been very severely affected for the past nine years. She has received care in a nursing home setting, hospital and now in her forties has around the clock care in the community.

Her difficulty swallowing started in 2015 whilst a resident in a nursing home. A speech and language therapist (SALT) diagnosed dysphagia, cause unspecified. A community dietitian visited and prescribed oral nutritional supplements (ONS). The patient was unable to tolerate these and became increasingly malnourished.

Although she was already significantly underweight, the primary health care team failed to recognize the severity of the situation. Over a seven week period she was almost completely unable to ingest any nutrition or hydration. As the situation deteriorated the patient was told that she would be sectioned under the Mental Health Act if she did not go to hospital. She reluctantly agreed to a voluntary admission.

Over a two week period in hospital she was intermittently given intravenous fluids, but no nutrition. She was screened using the malnutrition universal screening tool (MUST); her MUST score was 4 (2 or above denotes high risk of malnutrition [7]).

Her condition deteriorated further to the extent that she had to be admitted to a High Dependency Unit (HDU) because no Intensive Care Unit (ICU) bed was available, after which a Nasogastric Tube (NGT) was inserted. By this time, she was found to be suffering from a severe electrolyte imbalance, which further delayed the establishment of a feeding regime due to the risk of re-feeding syndrome.

Two months later with an established NGT feeding regime in place her MUST was still only 2.

The patient gained the strong impression that her doctors regarded her problems as psychological in origin and that she was being treated as if she was suffering from an eating disorder.

An NGT was sited prior to discharge. The patient was told another NGT would not be sited.

The tube remained in situ far longer than recommended until it became unusable. It was re-sited as an emergency in hospital. Subsequently the tube became blocked on several occasions and each time the patient had to attend the hospital as an emergency to have it replaced. This was especially concerning as there was no guarantee the tube would be re-sited, despite a clinical need.

In 2019 this situation improved after a Percutaneous Endoscopic Gastrostomy (PEG) was sited, and finally the patient had an improvement in her quality of life with the allocation of a Home Enteral Nutrition Service (HENS) dietician who made changes to the feed to ameliorate pain whilst being fed.

The patient still feels that there has been a failure to acknowledge her dysphagia. Today she has a normal BMI and continues to receive her preferred choice of care in the community.

### 3.2. Case 2

This female patient is in her 50s. She was diagnosed with ME following viral encephalitis in her twenties and has had very severe ME from the outset, being bed bound and needing around the clock care. She has required NGT feeding for over twenty five years. Following a diagnosis of intestinal failure two years ago she now requires parenteral feeding.

Within the first eighteen months of diagnosis, over a five month period, the patient started to experience swallowing difficulties complicated by facial paralysis leading to an inability to maintain adequate nutrition and hydration, and to significant weight loss. There was little intervention by primary care, but after five months of malnutrition the GP prescribed ONS. The nutritional difficulties and associated weight loss necessitated an emergency hospital admission.

NGT feeding was instigated following the intervention of a psychiatrist who stated that her nutritional difficulties were not psychological in origin. A SALT assessment confirmed the swallowing reflex was absent. Despite the diagnosis of dysphagia, a second psychiatrist told her that her inability to swallow was psychological. Three months later, with little weight gain, her discharge home was planned without an NGT. With family pressure she was finally sent home with an NGT in place.

For over twenty five years NGT feeding continued with the support of a HENS dietician. The GP arranged for an anesthetist to re-site the tube at home until this service was withdrawn. A private arrangement was made by the family to avoid travel to hospital, which would have been detrimental to the patient’s condition.

Recent abdominal surgery, complicated by adhesions, led to a bowel obstruction and another emergency admission to hospital due to extreme vomiting. Total Parenteral Nutrition (TPN) was commenced.

A peripherally inserted central catheter (PICC line) infection necessitated its removal and the patient was transferred to a specialist unit in another hospital. Despite the success of TPN, a trial of NGT feeding was enforced causing further weight loss with vomiting and abdominal pain. A barium meal showed gastroparesis and intestinal failure. An Nasojejunal Tube (NJT) was then sited but the patient experienced the same effects as with the NGT. Nutrition via TPN was then started, but despite this, further weight loss occurred. When this was brought to the attention of the medical team, they accused the patient of interfering with the pump despite this being physically impossible. By this point the patient’s BMI was 11.4. The family monitored the feed administered, and showed it was less than prescribed. A second opinion was requested by the family and Mast Cell Activation Disorder (MCAD) diagnosed. Medication was prescribed to treat this which stabilized and then increased the patient’s weight. On discharge from hospital her BMI was 13.8.

Currently, TPN is still being administered via a PICC line. Her BMI is 18.6. Unfortunately, there is currently no provision for community based TPN within the NHS, so administration and supply of the feed has had to be outsourced to a private company.

### 3.3. Case 3

This patient is a young adult with severe ME who had been cared for at home for over three years before becoming nutritionally deficient.

At home the patient was cared for in bed in a darkened room. Her family became concerned about her poor oral intake and the associated weight loss. Her mother tried repeatedly to get professional help in the community but it was not forthcoming; the patient required an emergency admission to hospital.

Once in hospital only IV fluids were given, with a delay of nine days before nutritional feed was given via a NGT. A SALT assessment failed to find a reason for the swallowing problems. An eating disorder was excluded. With the apparent lack of an organic reason for the situation, psychological problems and lack of motivation were suggested as a cause by hospital staff. Reluctance to allow long-term feeding via NGT was expressed, the reason cited being that she might become “dependent” on this form of treatment.

Accordingly, attempts were made to wean her off the NGT, in the form of longer waits between feeds. This was observed and challenged by the patient’s mother.

Two months into her admission the need for long term nutritional support in the community was accepted by the nutrition support team (NST). An NJT was inserted on the grounds that there was no domiciliary service for re-siting NGTs. She was then discharged and is maintaining a normal BMI while being cared for at home.

### 3.4. Case 4

This patient was diagnosed with ME as a child. Several years later the ME became very severe. She became virtually unable to eat and had severe nausea. An ME specialist recommended tube-feeding. The patient and her parents agreed to it. There was a lengthy delay because the local pediatricians refused it. Her inability to eat and the delay caused significant weight loss and the situation became life threatening. The patient was admitted to hospital where NGT feeding commenced. The local pediatricians claimed that she had anorexia nervosa and persuasive refusal syndrome and threatened admission to a psychiatric unit. A psychiatrist found that she did not have any psychiatric disorders. She was NGT fed for nine months before a PEG was sited.

### 3.5. Case 5

ME was diagnosed in childhood in this patient; she is now in her forties. She has had two episodes of enteral feeding, firstly in her thirties and more recently for a longer period of four years. She has needed emergency admissions to hospital on both occasions in order to access enteral feeding. As with the other cases there was no support in the community for the provision of NGT feeding.

At the first emergency admission, her BMI was 13.7 She was fed by NGT for three weeks, following which the NGT was removed prior to discharge. The patient felt she had been labelled as having an eating disorder. On discharge she was allocated an eating disorders dietician who alluded to the likelihood that she would be sectioned under the Mental Health Act if she lost more weight.

Following a move to a new area, a different consultant looked at her case and made a diagnosis of gastroparesis.

A worsening of her ME, a fall in her BMI to 14.8 and being barely able to consume any ONS led to another emergency admission.

On this occasion she was provided with a long-term plan. She was discharged with an NGT in situ and with the involvement of a HENS dietician. While in hospital she had been taught how to reinsert an NGT herself which she was then able to do at home, avoiding the need for further hospital admissions.

Working in conjunction with the HENS dietitian, the patient has progressed to modifying her diet to food which was more easily digested. She has been able to remove the NGT but still requires ONS to meet her calorific requirements. Her BMI is currently 20.5.

### 3.6. Summary

The experiences of all five participants share some strikingly similar features.

All had been allowed to become and remain severely malnourished and dehydrated. The experience was frightening and emotionally upsetting for the patients. The extent of the malnutrition could have further consequences, both short- and long-term. The re-introduction of nutrition poses an immediate risk of re-feeding syndrome [8] and there are possible longer-term consequences for the patients’ health status including poor wound healing, neurological damage, and osteoporosis [9].

Features emerging from these five cases include:

All were allowed to become severely malnourished and dehydrated to a life-threatening extent.

The inability to swallow in all cases was believed to be psychological in origin and psychiatrists became involved in all cases.

All five were considered to be suffering from anorexia nervosa; had this been the case it would have warranted tube feeding.

Two were advised that their enteral nutrition would be stopped, despite a clinical need.

Two were threatened that they would be sectioned under the Mental Health Act, if they did not eat and drink or if they lost weight again.

#### Scope and Limitations of Study

This case report documents the experiences of five people. While the number of cases reported here is small, our experience of supporting other severely affected ME patients has shown similar failures in a much larger number of cases.

## 4. Discussion

This series of cases demonstrate a common set of problems. The clinicians involved seemed unaware that severe ME can lead to serious problems maintaining adequate nutrition and hydration. Perhaps this is understandable, as many clinicians will only meet one or two cases of severe ME in their careers, and the subject is poorly taught at both undergraduate and postgraduate levels [5].

The doctors failed to recognize the severity of the malnutrition or to provide appropriate nutritional support in a timely manner [10]. Each case developed life-threatening problems as a result and were only saved by the late introduction of some form of nutritional support. Clinical inertia was evident throughout. In respect of the repeated finding that patients were wrongly regarded as having an eating disorder as a cause for their nutritional problems, it is lacking in logic for the doctors concerned not to have treated this on its own merit. Tube feeding, with or without a court order, is frequently resorted to in cases of an eating disorder. Either the doctors were not serious in making this diagnosis or they were somehow generally prejudiced against the patients on account of their being cases of ME/CFS. In each case, the doctors resorted to making inappropriate psychological diagnoses without positive evidence of psychopathology.

NICE Clinical Guideline 53 on ME/CFS, lists the nutritional problems which may be experienced by a person with severe ME/CFS and also notes the possible need for tube feeding in patients with severe ME:

“this may include the use of tube feeding, if appropriate [3].”

NICE suggest using Clinical Guideline 32 ‘Nutrition support for adults: oral nutrition support, enteral feeding and parenteral nutrition’ [11] for any patients who are nutritionally compromised. This document is referenced in CG53 [3]. Each of the patients met the criteria for enteral feeding set out in CG32. Given the nutritional status of the patient, the clinicians should have followed the NICE guidance.

Case 2 highlights an important issue. If a patient is failing to respond to enteral feeding, the possibility of MCAD needs to be considered. This is a recognized complication of severe ME and effective treatment exists in the form of oral cromoglycate and antihistamines. It has probably contributed to several deaths of severe ME sufferers.

In every case, the most positive improvement in their management came about as the result of the allocation of a named HENS dietician whose advanced training in enteral nutrition enabled them to make changes to the patient’s diet. In one case it enabled the patient to get to a healthy weight using enteral nutrition whilst making changes to the oral nutrition such that enteral feeding is now no longer required. In another case, dietary changes ameliorated suffering. All patients felt supported by their HENS dietician. For patients with very severe ME connecting with a knowledgeable healthcare professional who does domiciliary visits is very important. Such a policy would reduce the need for hospital admissions which would be to the benefit of all. All patients with very severe ME should be allocated a HENS dietician as soon as nutritional difficulties become apparent.

An early warning system needs to be put in place for patients with severe ME so that when they or their representatives become aware of the development of problems with oral intake prompt action is taken and tube feeding started thereby avoiding undernutrition in patients with very severe ME. Early intervention in the form of tube feeding has been shown to be beneficial in patients with severe ME [1].

Patients with very severe ME are bedridden and require around the clock care. They are best cared for at home where the environment can be adapted to best meet their needs. These patients will have extreme sensitivity to noise and light, such that they need to be cared for in a darkened room. People with very severe ME invariably report travel to hospital and the hospital environment significantly exacerbates their condition. If an admission to hospital is necessary, and this should only be done for emergency treatment, they will require admission directly into a side room and to be cared for by a small number of staff who understand ME as an organic illness.

For the patient with very severe ME, it appears to be common practice for Clinical Commissioning Groups (CCGs) to adopt a ‘re-site in hospital’ policy despite a large study showing that with protocols in place trained nurses in the community can identify the position of NGT’s correctly without the need for hospital attendance [12].

Nonetheless it is stated:

‘Local protocols should address the clinical criteria that permit enteral feeding [13].’

None of the participants were offered NGT re-sites at home, instead they went to significant lengths to avoid trips to hospital if at all possible; re-siting their own NGTs or opting to have NJTs or PEGs. A constructive change would an implementation of national guidelines allowing NGT re-sites to be carried out in the community by appropriately trained professionals. A community-based service could bring potential savings to the NHS and certainly benefit patients with very severe ME. The treatment of serious undernutrition issues in ME needs to be included in national and local guidelines for use by health care professionals.

## 5. Conclusions

To remedy the problems identified in this survey, the most important first step remains to improve medical education for healthcare professionals regarding the fact that severe ME can cause nutritional problems, and that these may require early intervention with tube feeding. Progress has been made in that a Continuing Professional Development (CPD) Module on ME has been developed [14] and launched in May 2020. The uptake was very poor with fewer than two thousand clinicians taking the module to date. Medical education around ME needs to be made part of the core curriculum for undergraduate students and should also be included in postgraduate education. It is necessary for the clinician to recognize ME/CFS as an organic illness. It can only be hoped that the new NICE Guidelines aid clinicians’ understanding and provide guidance on dealing with nutritional problems such as those described in this series. 

## Data Availability

The data presented in this study are available on request from the corresponding author.

## Supplementary Materials

The following are available online at https://www.mdpi.com/article/10.3390/healthcare9040459/s1. Enteral and parenteral feeding questionnaire.
