# Supplementary material for: Life-Threatening Malnutrition in Very Severe ME/CFS
Source: Healthcare (Basel). 2021 Apr 14;9(4):459. doi: 10.3390/healthcare9040459 (PMC8070213; doi:10.3390/healthcare9040459)
Supplement: Supplementary file 1 [file healthcare-09-00459-s001.pdf]

ENTERAL AND PARENTERAL FEEDING QUESTIONNAIRE

Are You:- [Circle all that apply]

NG Fed go to **Section 1**

NJ Fed go to **Section 2**

PEG Fed go to **Section 3**

PEJ Fed go **to Section 4**

On TPN go to **Section 5**
Everyone needs to complete Section 6

Section 1 NG Fed members only

Age ___________

How long did you have ME before being NG fed ?__________________________

Reasons for NG Feeding ____

____

How long have you been NG fed? _______________________

Hospital ____

Consultant Gastroenterologist ____

Who manages your tube on a daily basis i.e., flushing/ aspirating?

____

Who oversees the care of the tube and your nutrition in the community?

____

When the tube needs replacing where does this take place?

____

Is there a plan in place for the replacement e.g. elective resiting?

____

<u>Section 2 NJ Fed members only</u>

Age ----------------

How long did you have ME before being NJ fed? ____________________

Reasons for NJ Feeding ____

____

How long have you been NJ fed? _____________

Hospital ____

Consultant Gastroenterologist _______________________________________

Who manages your tube on a daily basis e.g. flushing?

____

Who oversees the care of the tube and your nutrition in the community?

____

When the tube needs replacing where does this take place?

____

Is there a plan in place for the replacement e.g. elective re-siting? ______

Are you NJ fed due to gastroparesis? _____________

Have you been tested for / diagnosed with Mast Cell Activation Disorder ? If "YES" to the above, circle as appropriate

<u>Section 3 PEG Fed members only</u>

Age __________

Hospital ____

Consultant Gastroenterologist ____

How long did you have ME for before any form of tube feeding commenced?__________

How long did you have ME before being PEG fed? ________________________

Were you NG fed before being PEG fed? _____________________

If so, why was the decision made to site a PEG? ___________________________________

____

Reasons for PEG Feeding _______________________________________

How long have you been PEG fed? ___________________________________

Who manages your tube on a daily basis e.g. flushing/ aspirating?

____

Who oversees the care of the tube and your nutrition in the community?

____

Section 4 PEJ Fed members only

Age __________

Hospital ____

Consultant Gastroenterologist ____

How long did you have ME for before any form of tube feeding commenced?__________

How long have you been PEJ fed? _______________

What other forms of tube feeding were tried prior to PEJ feeding?___________________

Why and by whom was it decided you needed a PEJ?______________________________

____

Who manages your tube on a daily basis e.g. flushing?

____

Who oversees the care of the tube and your nutrition in the community?

____

Are you PEJ fed due to gastrointestinal failure? __________________

Have you been tested for / diagnosed with Mast Cell Activation Disorder? If 'YES' to the above, circle as appropriate

Section 5 Total Parenteral Nutrition

Age __________

Hospital ____

Consultant Gastroenterologist ____

How long did you have ME for before any form of tube feeding commenced?__________

How long have you been on TPN? ____

What other forms of tube feeding were tried prior to TPN?_________________________

How long were they tried for and why were they stopped?_________________________

____

Were you allowed to become underweight whilst different types of tube feeding were tried?

____

Who manages your central line on a daily basis?

____

Are you on TPN due to gastrointestinal failure? ___________________

Have you been tested for / diagnosed with Mast Cell Activation Disorder? If "YES" to the above, circle as appropriate

Section 6 All enteral / parenteral fed members

Have you been assessed by SALT? _______________

Were you diagnosed with an unsafe swallow? __________________

Were you allowed to become underweight prior to tube feeding commencing? ________

Was your inability to eat ever considered to be anorexia nervosa? _____________

Were you ever threatened with sectioning prior to tube feeding commencing? ______

Do you feel your health has improved by being tube fed? _____________

Any other relevant information: - ____

Thank you for taking the time to complete the questionnaire.

June 2019
